# Eye Movements during Silent and Oral Reading in a Regular Orthography: Basic Characteristics and Correlations with Childhood Cognitive Abilities and Adolescent Reading Skills

**DOI:** 10.1371/journal.pone.0170986

**Published:** 2017-02-02

**Authors:** Magdalena Krieber, Katrin D. Bartl-Pokorny, Florian B. Pokorny, Dajie Zhang, Karin Landerl, Christof Körner, Franz Pernkopf, Thomas Pock, Christa Einspieler, Peter B. Marschik

**Affiliations:** 1 Research Unit iDN–Interdisciplinary Developmental Neuroscience, Institute of Physiology, Center for Physiological Medicine, Medical University of Graz, Graz, Austria; 2 BEE-PRI: Brain, Ears & Eyes–Pattern Recognition Initiative, BioTechMed-Graz, Graz, Austria; 3 Machine Intelligence & Signal Processing Group, Technische Universität München, Munich, Germany; 4 Developmental Psychology, Institute of Psychology, University of Graz, Graz, Austria; 5 Cognitive Psychology & Neuroscience, Institute of Psychology, University of Graz, Graz, Austria; 6 Signal Processing and Speech Communication Laboratory, Graz University of Technology, Graz, Austria; 7 Institute for Computer Graphics and Vision, Graz University of Technology, Graz, Austria; 8 Center of Neurodevelopmental Disorders (KIND), Department of Women’s and Children’s Health, Karolinska Institutet, Stockholm, Sweden; University of Leicester, UNITED KINGDOM

## Abstract

The present study aimed to define differences between silent and oral reading with respect to spatial and temporal eye movement parameters. Eye movements of 22 German-speaking adolescents (14 females; mean age = 13;6 years;months) were recorded while reading an age-appropriate text silently and orally. Preschool cognitive abilities were assessed at the participants’ age of 5;7 (years;months) using the *Kaufman Assessment Battery for Children*. The participants’ reading speed and reading comprehension at the age of 13;6 (years;months) were determined using a standardized inventory to evaluate silent reading skills in German readers (*Lesegeschwindigkeits- und -verständnistest für Klassen 6–12*). The results show that (i) reading mode significantly influenced both spatial and temporal characteristics of eye movement patterns; (ii) articulation decreased the consistency of intraindividual reading performances with regard to a significant number of eye movement parameters; (iii) reading skills predicted the majority of eye movement parameters during silent reading, but influenced only a restricted number of eye movement parameters when reading orally; (iv) differences with respect to a subset of eye movement parameters increased with reading skills; (v) an overall preschool cognitive performance score predicted reading skills at the age of 13;6 (years;months), but not eye movement patterns during either silent or oral reading. However, we found a few significant correlations between preschool performances on subscales of sequential and simultaneous processing and eye movement parameters for both reading modes. Overall, the findings suggest that eye movement patterns depend on the reading mode. Preschool cognitive abilities were more closely related to eye movement patterns of oral than silent reading, while reading skills predicted eye movement patterns during silent reading, but less so during oral reading.

## 1. Introduction

It is a common strategy of beginning readers to focus on oral reading, i.e. grapheme-phoneme-conversion and reading of chunked units, such as syllables and words. Verbalization of written content allows the teacher to monitor the reading process and prevent the reader from skipping complex words. With time, silent reading becomes increasingly important until eventually it replaces oral reading as preferred reading mode as we become competent readers (e.g., [1–3]).

Although there is a substantial body of literature comparing silent to oral reading with respect to speed/efficiency or text comprehension (e.g., [3–8]), the majority of eye movement studies are focused on characteristics of silent reading (e.g., [9–13]; for a review, see [14]). Comparably little emphasis has been put on ocular behavior during oral reading (e.g., [15–22]) and on the comparison of intraindividual eye movements during silent and oral reading (e.g., [23–27]). Quite apart from the technical challenge of recording high-resolution eye movement data during oral reading, this lack might be caused by the assumption that, apart from the apparent difference in verbalization, both reading modes are based on the same underlying processes and strategies (e.g., [2,21–24]).

Systematic reviews of eye movement studies investigating either silent or oral reading in adults have reported some significant differences between these two reading modes: fixation duration was about 50 ms longer when reading orally (275–325 ms vs. 225–250 ms for silent reading); saccadic amplitudes were reported to be shorter (6–7 letters for oral reading vs. 7–9 letters for silent reading); the number of regressions was higher (e.g., [14,28,29]). In addition, Huestegge [30] reported about an interaction between phonetic properties and eye movements dependent on the reading mode: vowel length interacted with gaze duration during silent reading, but not during oral reading. However, the above mentioned studies [14,28–30] did not compare eye movements across reading modes intraindividually.

In the late 1930s, Anderson and Swanson [23] were the first to examine differences in eye movements during silent and oral reading within subjects. In a sample of university students with good and poor reading skills, they found (a) eye movement parameters to reflect silent reading to be faster than oral reading; (b) a positive association between silent and oral reading performances, with somewhat higher correlation coefficients in poor compared to good readers; and (c) a smaller difference between the two reading modes in less skilled readers than in highly skilled ones.

In more recent years, the parafoveal preview benefit (i.e. increased reading efficiency due to parafoveal preprocessing of upcoming words; for reviews, see e.g., [14,28]) was considered to vary across reading modes. Using either the moving window paradigm (i.e. the amount of parafoveally visible information is manipulated through the use of a gaze-contingent moving text window; [24]) or the boundary paradigm (i.e. parafoveally visible information is manipulated through the change of a preview stimulus to a target stimulus as soon as the participant’s gaze crosses an invisible boundary; [25]), findings indicated that parafoveally available information increased reading speed in both reading modes. However, this effect was more significant and consistent in silent reading.

All studies mentioned above included adult, i.e. relatively skilled readers. As reading processes differ according to competence, findings from adults may not apply to developing readers. Only in 2014 did Vorstius and colleagues [26] focus on developing readers’ eye movements when silently and orally reading sentences with simple grammatical structures. In their sample of English-speaking first- to fifth-graders, the authors found oral reading to be associated with longer fixation and refixation durations, more frequent refixations, and a higher probability to fixate a word. Interestingly, besides shorter saccades, Vorstius and colleagues [26] found shorter rereading times and fewer interword-regressions in oral than in silent reading, which the authors attributed to the effort to maintain an optimal eye-voice span (i.e. the distance the eyes are ahead of the voice; e.g., [15,20,22]).

Although most reading research is based on English-speaking cohorts, a number of studies have focused on eye movements in readers of more regular orthographies, such as German or Italian (e.g., [10,17,20,30–36]). Orthographic consistency is known to influence reading development (e.g., [37,38]) and the spatial and temporal characteristics of eye movements in both developing and skilled readers (e.g., [39,40]). To the best of our knowledge, only one study includes an intraindividual comparison of eye movements across reading modes in readers of a regular orthography [27]. However, the main aim of Paeglis and colleagues [27] in analyzing the eye movements of six Latvian participants was to compare performances of three different eye-tracking systems with varying technical specifications, rather than to investigate differences between reading modes.

As of yet, no thorough within-subject comparison of silent and oral reading in a regular orthography has been reported in the eye-tracking literature. Previous research pointed out interactions between orthographic depth (i.e. the consistency of grapheme-phoneme-conversion) and oculomotor control (e.g., [39,40]). We therefore examined the differences and relation between basic, well researched eye movement parameters across silent and oral reading specific to German, which has a highly consistent orthography. With respect to the findings of Vorstius and colleagues [26], and studies investigating the eye-voice span during oral reading (e.g., [15,20,22]), we intended to characterize the influence of the need to keep a constant span between eyes and voice during oral reading on the intraindividual consistency of eye movement patterns (i.e. the correlations between eye movement parameters during silent and oral reading). This refers to Anderson and Swanson’s assumption [23] that superior reading performances blur during oral reading.

Previous research reported stable effects of reading proficiency on oculomotor control during silent reading (e.g., [9,10,34,35,41]), but less is known on this relation during oral reading (e.g., [17,20–22]). As articulation is a limiting factor to the maximum reading speed, the difference between these two reading modes is suspected to increase with reading proficiency and skilled readers generally favor silent reading over oral reading (e.g., [1–3,23]). The first and so far last attempt to delineate the relation between reading skills and the difference in the oculomotor control during silent and oral reading focused on a rather small set of eye movement parameters in English readers [23]. We aimed to characterize distinct influences of reading skills on (a) oculomotor control during both silent and oral reading, and (b) the difference in eye movements across reading modes using a state-of-the-art high-resolution eye-tracking device and taking into account a larger number of basic eye movement measures.

Longitudinal studies focusing on associations between cognitive and reading/writing abilities revealed interesting associations between different intelligence-related skills and a number of speech-language-related achievements for both good and poor readers (e.g., [6,42–44]). In addition to the objectives outlined above, we aimed to determine the predictive power of preschool cognitive abilities on reading skills and oculomotor control during silent and oral reading in adolescence.

More precisely, we addressed the following questions: (i) To what extent do eye movement patterns reflect the differences between silent and oral reading strategies in adolescent readers of a regular orthography? (ii) Do eye movement parameters differ with respect to the intraindividual consistency across reading modes? (iii) How are reading skills associated with eye movements during silent and oral reading? (iv) How are reading skills related to differences in eye movement parameters between silent or oral reading? (v) Do preschool cognitive abilities predict reading skills and oculomotor control during silent and oral reading in adolescence?

## 2. Methods

### 2.1. Participants

In 1998, 62 newborns and their German-speaking families resident in or near Graz (Austria) were recruited for the *Developmental Physiology & Developmental Neuroscience 2* study on neuromotor, cognitive, and speech-language development. At the age of 13;6 (years;months), 23 participants of the initial cohort agreed to volunteer for this follow-up. Due to esotropia, one participant was excluded from the analyses; the remaining 22 participants (14 females; *M* = 13;6 years;months of age; *SD* = 2 months) had normal or corrected-to-normal vision (for further details on the sample, see e.g., [44–46]).

The study was approved by the Institutional Review Board of the Medical University of Graz (Austria). Adolescents were supplied with age-appropriate information on the purpose of the study and the experimental tasks. In addition to the adolescents’ assent, written informed consent was obtained from their parents.

### 2.2. Assessment of preschool cognitive abilities

Preschool cognitive abilities were assessed at the participants’ age of 5 years and 7 months using the German version of the *Kaufman Assessment Battery for Children* (K-ABC; [47]), a standardized assessment to evaluate intellectual abilities in children from 2;6–12;5 (years;months of age). The K-ABC consists of four subscales (i.e. sequential processing, simultaneous processing, mental processing composite, achievement) and a total of 16 subtests. We focused on the seven of ten K-ABC subtests (comprising the mental processing composite score) applicable in children aged 5;0–5;11 (years;months), which added up to a test duration of approximately 50–60 minutes.

### 2.3. Assessment of reading skills

Reading skills were determined using the *Lesegeschwindigkeits- und -verständnistest für Klassen 6–12* (LGVT [Reading Speed and Reading Comprehension Test for Grades 6–12]) [48], a standardized inventory to assess silent reading speed and reading comprehension in German-speaking 12- to 18-year-olds. The LGVT consists of a single text with 1727 words, from which both the reading speed and reading comprehension scores are calculated. The number of words read within 4 minutes equals the reading speed score. To evaluate reading comprehension 23 multiple choice questions have to be answered.

### 2.4. Materials

Participants were instructed to read an age-appropriate German school-book text about the origin of life ([49]; pages 7–8). The passage consisted of 13 sentences and a total of 150 words (average word length = 6.4 graphemes). We used a black monospaced font (Courier New) with the font size corresponding to a letter width of 0.5°; text lines were double-spaced. Due to varying sentence lengths, each page (11 in total) consisted of two to four lines.

### 2.5. Apparatus and procedure

The study was conducted at iDN’s BRAIN*tegrity* lab at the Institute of Physiology, Medical University of Graz (Austria). A tower-mounted, video-based eye-tracking system (iView X Hi-Speed; SensoMotoric Instruments; Teltow, Germany) was used to record binocular eye movements with a sampling rate of 500 Hz. In the silent reading mode, we used forehead and chin rests to restrict head movements. The chin rest was removed to facilitate articulation for oral reading (Speak-Aloud^TM^ kit; SensoMotoric Instruments; Teltow, Germany). Additionally, we recorded the participants’ verbalizations using a Mc Crypt BM-700 studio microphone.

The eye-tracker and a 22-inch stimulus monitor (1680 × 1050 pixels) were positioned on a height-adjustable table. Participants were seated at a distance of 90 cm to the monitor, resulting in a screen size of 29.5° (horizontally) × 18.7° (vertically). The participants’ line of sight was approximately 10 cm above the monitor’s center.

Prior to the reading task, each participant underwent a 13-point calibration routine followed by a four-point quality check. Gaze position errors were generally below 0.4°.

The school-book text (see section 2.3.) was presented in two separate runs. First, participants were instructed to read silently, comprehensively and carefully. After a break of approximately 30 minutes (during which the setup was adapted for oral reading and the LGVT as well as a number of neuromotor assessments were conducted) participants reread the text, but were now asked to read aloud and articulate clearly and understandably. We did not instruct participants how to overcome reading errors (e.g., omitting syllables or words). The order of reading modes was the same for all participants (see section 4.1. on limitations of the study design).

### 2.6. Data analysis

Data from the dominant eye was extracted using SMI BeGaze (SensoMotoric Instruments; Teltow, Germany). We analyzed eleven common spatial and temporal eye movement parameters: (1) total word/text reading time; (2) first fixation duration; (3) average fixation duration; (4) gaze duration (total time spent fixating words during first-pass reading); (5) refixation time (gaze duration–first fixation duration); (6) rereading time (total word reading time–gaze duration); (7) total number of fixations per word; (8) total number of saccades; (9) total number of regressions; (10) percentage of regressions; and (11) mean saccadic amplitude.

Event detection parameters were (a) a minimum peak velocity of 40°/s and a minimum duration of 22 ms for saccades; and (b) a minimum duration of 50 ms for fixations.

We used paired-samples *t*-tests to compare eye movements across reading modes. Pearson’s product-moment correlation coefficients and linear regression analyses were used to assess associations between and predictability of eye movement parameters. To compare correlation coefficients between two overlapping pairs of variables, we applied Steiger’s *Z*-test [50]. All analyses were performed using IBM SPSS Statistics 22 (SPSS Inc., Chicago, IL). The level of significance was set at 5%. During oral reading, one participant exhibited a mean gaze duration of 565 ms (i.e. more than three standard deviations above the sample’s mean); to ensure comparability of the results we excluded this participant from statistical analyses, resulting in a final sample size of n = 21.

## 3. Results

### 3.1. A comparison between silent and oral reading

Means and standard deviations for each eye movement parameter for silent and oral reading are given in the middle columns of Table 1. The results of the paired-samples *t*-tests (right column of Table 1) show the reading mode to have had a significant effect on the eye movement parameters assessed (mean saccadic amplitude: *p* < .01; all other *p*s < .001), except for the percentage of regressions, which approached significance (*p* = .054). Compared with oral reading, when reading silently participants (a) fixated less frequently and for shorter periods of time, (b) made fewer saccades and regressions, and (c) made saccades of larger amplitudes. These results suggest that reading was faster and appeared less demanding during silent as compared to oral reading.

**Table 1:** Descriptive statistics and paired-samples *t*-tests.

	Silent Reading		Oral Reading		Difference (oral–silent reading)		*t*-test
	*Mean*	*SD*	*Mean*	*SD*	*Mean*	*SD*	*t(20)*
**Total text reading time (ms)**	56481	12673	79196	14379	22715	14781	-7.04***
**Total word reading time (ms)**	377	84	528	96	151	99	-7.04***
**First fixation duration (ms)**	213	24	228	29	15	16	-4.23***
**Average fixation duration (ms)**	207	23	225	26	18	20	-4.21***
**Gaze duration (ms)**	323	67	389	41	66	66	-4.58***
**Refixation time (ms)**	109	53	160	38	51	57	-4.07***
**Rereading time (ms)**	54	33	139	81	85	80	-4.89***
**Number of fixations per word**	1.24	0.22	1.62	0.27	0.38	0.28	-6.27***
**Total number of saccades**	143.5	32.4	183.2	29.5	39.7	26.2	-6.94***
**Total number of regressions**	31.0	12.6	48.6	19.4	17.6	18.8	-4.29***
**Percentage of regressions (%)**	18	8	21	6	3	6	-2.05^+^
**Mean saccadic amplitude (°)**	3.30	0.65	2.99	0.43	-0.31	0.45	3.08**

***p < .001

**p < .01

^+^*p* = .054.

### 3.2. Intraindividual consistency of eye movements across reading modes

For five of eleven eye movement parameters we found significant correlations between silent and oral reading, including first fixation duration (*r* = .83, *p* < .001); average fixation duration (*r* = .66, *p* < .01); total number of saccades (*r* = .64, *p* < .01); percentage of regressions (*r* = .66, *p* < .01); and mean saccadic amplitude (*r* = .73, *p* < .001). The coefficients for total text reading time (*r* = .41, *p* = .066), gaze duration (*r* = .33, *p* = .138), refixation time (*r* = .25, *p* = .276), rereading time (*r* = .21, *p* = .357), the number of fixations per word (*r* = .38, *p* = .090), and the total number of regressions (*r* = .37, *p* = .100) failed to reach significance. These results show that eye movements differ with respect to their intraindividual consistency between silent and oral reading, as a substantial number of eye movement parameters was not significantly correlated across reading modes.

### 3.3. How do reading skills influence eye movements during silent and oral reading?

Although LGVT reading speed and comprehension were closely related (*r* = .76, *p* < .001) we decided to use multiple linear regression analyses to determine the influence of both predictors on eye movements during silent and oral reading (Table 2). Due to the shared variance of reading speed and reading comprehension we expected the validity of beta weights to be limited and additionally referred to zero-order correlations to support the interpretation of results.

**Table 2:** Linear regression parameters and zero-order correlations for eye movement parameters as predicted by LGVT reading speed and reading comprehension for silent and oral reading.

	Silent reading					Oral reading				
		*Reading speed*		*Reading comprehension*			*Reading speed*		*Reading comprehension*	
	R^2^	Β	r	β	r	R^2^	β	R	β	r
**Total text reading time**	.46**	-.72*	-.68	.06	-.49	.09	-.07	-.25	-.24	-.29
**First fixation duration**	.26^+^	-.70*	-.46	.31	-.22	.12	-.41	-.34	.09	-.22
**Average fixation duration**	.29*	-.78*	-.47	.40	-.19	.17	-.56	-.38	.23	-.19
**Gaze duration**	.46**	-.81**	-.67	.19	-.43	.13	.11	-.22	-.43	-.35
**Refixation time (ms)**	.39*	-.69*	-.62	.09	-.43	.13	.43	.02	-.54	-.21
**Rereading time (ms)**	.17	-.21	-.39	-.23	-.39	.04	-.13	-.18	-.06	-.17
**Number of fixations per word**	.47**	-.55*	-.68	-.17	-.59	.09	.24	-.10	-.44	-.26
**Total number of saccades**	.50**	-.49^+^	-.68	-.26	-.63	.20	.19	-.25	-.58^+^	-.43
**Total number of regressions**	.02	-.22	-.05	.23	.06	.03	.21	.16	-.05	.10
**Percentage of regressions**	.08	-.02	.20	.29	.28	.10	.08	.27	.24	.30
**Mean saccadic amplitude**	.60***	.72**	.77	.07	.61	.36*	-.09	.42	.66*	.60

***p < .001

**p < .01

*p < .05

^+^.05 < *p* < .10.

In case of silent reading, the coefficients of determination for seven of eleven regression models were significant: differences in LGVT reading skills significantly explained a variance of 29% to 60% in total text reading time (*F*(2,18) = 7.66, *p* < .01), average fixation duration (*F*(2,18) = 3.68, *p* < .05), gaze duration (*F*(2,18) = 7.60, *p* < .01), refixation time (*F*(2,18) = 5.76, *p* < .05), the number of fixations per word (*F*(2,18) = 8.10, *p* < .01), the total number of saccades (*F*(2,18) = 8.88, *p* < .01), and saccadic amplitudes (*F*(2,18) = 13.30, *p* < .001). A similar, but non-significant statistical trend was observed for first fixation duration (*F*(2,18) = 3.10, *p* = .070). In contrast, reading speed and comprehension did not predict reading performances with regard to rereading times and leftward eye movements (total number of regressions and percentage of regressions; all *F*s < 2.00, all *p*s > .05). Beta weights consistently indicated that reading speed was a better predictor than reading comprehension in each of the significant models for silent reading. Zero-order correlation coefficients revealed that both predictors shared variance with the eye movement parameters assessed, though reading comprehension could not additionally contribute to the proportion of variance explained by reading speed. However, the associations between reading speed and the criterion variables did not significantly differ from those observed for reading comprehension (all *Z*s < 1.96, all *p*s > .05).

When reading orally, reading skills reliably predicted saccadic amplitudes, with the regression model accounting for 36% of variance (*F*(2,18) = 5.01, *p* < .05). In line with the results for silent reading, the associations between the predictors and the criterion variable were not significantly different (*Z* = 1.32, *p* > .05). The remaining ten parameters could not be explained by means of LGVT reading skills (all *F*s < 2.30, all *p*s > .05).

Taken together, these findings suggest that an increase in reading skills decreases the time needed to process written content during silent reading, but less so during oral reading (see section 4.1. on limitations of the study).

In a second step, we compared the correlation coefficients between LGVT reading skills and eye movement parameters for silent and oral reading. During silent reading, LGVT reading speed was more closely related to total text reading time (*Z* = 2.03, *p* < .05), gaze duration (*Z* = 1.98, *p* < .05), refixation time (*Z* = 2.43, *p* < .05), the number of fixations per word (*Z* = 2.55, *p* < .05), the total number of saccades (*Z* = 2.55, *p* < .05), and saccadic amplitudes (*Z* = 2.71, *p* < .01) than during oral reading (all other *Z*s < 1.96, *p*s > .05). The associations between LGVT reading comprehension and eye movement parameters did not differ between reading modes (all *Z*s < 1.50, all *p*s > .05).

### 3.4. Moderating effects of reading skills on the difference in eye movements between silent and oral reading

To estimate the predictive power of reading skills for differences between silent and oral reading, data were entered into multiple linear regression models with LGVT reading speed and reading comprehension as predictors and the intraindividual difference of each eye movement parameter as criterion (Table 3). For gaze duration (*F*(2,18) = 5.48, *p* < .05), refixation time (*F*(2,18) = 6.98, *p* < .01), the total number of saccades (*F*(2,18) = 5.28, *p* < .05), and saccadic amplitudes (*F*(2,18) = 15.89, *p* < .001), the regression models were significant, with LGVT reading skills accounting for 37% to 64% of variance in the difference between silent and oral reading. The model for the number of fixations per word was marginally significant (*F*(2,18) = 2.80, *p* = .088). As for the remaining parameters, differences between silent and oral reading did not vary with LGVT reading skills (all *F*s < 2.00, all *p*s > .05).

**Table 3:** Linear regression parameters and zero-order correlations for differences in eye movement parameters between silent and oral reading as predicted by LGVT reading speed and reading comprehension.

*Difference between silent and oral reading in*:		*Reading speed*		*Reading comprehension*	
	R^2^	β	r	β	r
**Total text reading time**	.15	.55	.34	-.28	.14
**First fixation duration**	.05	.32	.09	-.30	-.06
**Average fixation duration**	.02	.20	.07	-.17	-.02
**Gaze duration**	.38*	.89**	.54	-.46	.21
**Refixation time (ms)**	.44**	.93**	.59	-.44	.26
**Rereading time (ms)**	.00	-.05	-.02	.03	.00
**Number of fixations per word**	.24^+^	.67*	.45	-.30	.21
**Total number of saccades**	.37*	.82*	.57	-.33	.30
**Total number of regressions**	.06	.36	.20	-.21	.07
**Percentage of regressions**	.01	.11	.02	-.12	-.04
**Mean saccadic amplitude**	.64***	-1.13***	-.72	.53*	-.32

***p < .001

**p < .01

*p < .05

^+^*p* = .088.

Although beta weights might have been affected by the shared variance of LGVT reading speed and comprehension, zero-order correlation coefficients showed for each significant model, except for the number of saccades (*Z* = 1.87, *p* > .05), that reading speed tended to be more closely related to differences between reading modes than reading comprehension (gaze duration: *Z* = 2.21, *p* < .05; refixation time: *Z* = 2.28, *p* < .05; saccadic amplitude: *Z* = 3.01, *p* < .01).

These results indicate that differences between silent and oral reading–including gaze duration, refixation time, the total number and length of eye movements–increase with reading skills, that is, differences are more pronounced in better readers than they are in less-skilled readers. However, a significant effect was observed for only four of eleven models, suggesting that for the majority of eye movement parameters, differences between reading modes could not be predicted by LGVT reading skills.

### 3.5. Do preschool cognitive abilities predict reading skills and oculomotor control during silent and oral reading in adolescence?

At the age of 5;7 (years;months), the mean standardized scores of the K-ABC subscales were the following: sequential processing scale: *M* = 102.7 (*SD* = 16.6); simultaneous processing scale: *M* = 110.3 (*SD* = 11.4); mental processing composite scale: *M* = 107.2 (*SD* = 11.6). We used linear regression analyses to estimate the predictive power of the participants’ mental processing composite score on LGVT reading skills and eye movement parameters during silent and oral reading at the age of 13;6 (years;months).

Performances on the mental processing composite scale were significantly predictive of both LGVT reading speed and comprehension at the age of 13;6 (years;months), with the regression models accounting for 27% and 32% of variance in the criterion variables, respectively (LGVT reading speed: *F*(1,18) = 6.75, *β* = .52, *p* < .05; LGVT reading comprehension: *F*(1,18) = 8.60, *β* = .57, *p* < .01).

**Fig 1:**
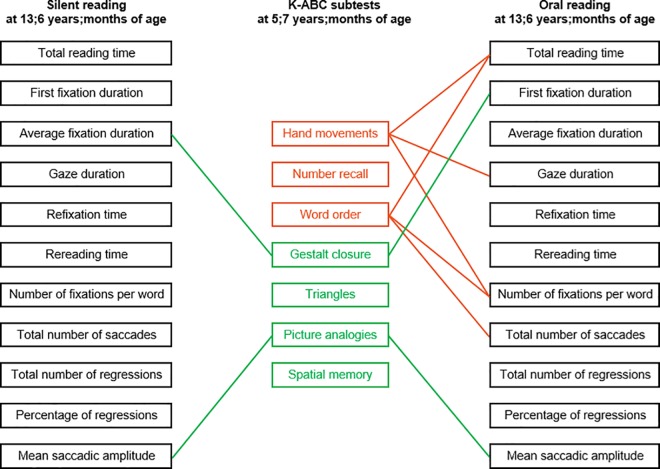
Correlations between preschool cognitive performance and eye movement parameters of silent and oral reading Pearson’s product-moment correlations between seven subtests of the K-ABC [47] corresponding to the sequential processing scale (orange) and the simultaneous processing scale (green) and eye movements during silent and oral reading. Green and orange lines indicate significant correlations (*p* < .05), suggesting that better performances on the K-ABC scales were associated with more efficient oculomotor performances.

In contrast to these results, preschool cognitive abilities did not significantly predict oculomotor parameters (Table 4; all *F*s < 3.80, all *p*s > .05), but we found a small number of eye movement parameters to be significantly correlated with the participants’ scores on the subtests of sequential and simultaneous processing (Fig 1; *r*s ranging from ±.44 to .53). Overall, the results indicate that preschool cognitive performances were only marginally associated with eye movement patterns of silent reading, while the association was somewhat higher for oral reading.

**Table 4:** Linear regression parameters for eye movement parameters as predicted by K-ABC mental processing composite scores.

	*Silent reading*		*Oral reading*	
	R^2^	β	R^2^	β
**Total text reading time**	.05	-.22	.15^+^	-.39^+^
**First fixation duration**	.14	-.38	.17^+^	-.42^+^
**Average fixation duration**	.13	-.37	.08	-.29
**Gaze duration**	.05	-.23	.04	-.20
**Refixation time (ms)**	.01	-.12	.01	.11
**Rereading time (ms)**	.01	-.09	.14	-.38
**Number of fixations per word**	.04	-.19	.11	-.33
**Total number of saccades**	.04	-.21	.09	-.30
**Total number of regressions**	.00	.03	.05	-.23
**Percentage of regressions**	.01	.09	.01	-.08
**Mean saccadic amplitude**	.11	.33	.06	.25

^+^.05 < *p* < .10.

## 4. Discussion

While many studies have investigated various aspects of eye movements during reading, the difference between silent and oral reading has received only little attention as of yet (e.g., [23–27]). The assessment discussed in this paper was part of a longitudinal study (aspects of reading development are presented in Bartl-Pokorny et al. [45] and Krieber et al. [46]). The main aim here was to highlight differences between silent and oral reading in adolescent readers of German, a regular orthography.

In general, our findings were in line with Anderson and Swanson [23], Vorstius and colleagues [26], and reviews by Rayner [14,28], and Schotter and Rayner [29]. We found (i) the eye movement patterns of adolescent German readers to differ between silent and oral reading both spatially and temporally. This has been discussed from a cognitive psychologist’s perspective, that for example increased fixation durations during oral reading may be related to dual-response costs when simultaneously executing oculomotor and articulatory movements [51]. Our results for each parameter revealed significant differences between the two reading modes (except for an only marginally significant difference for the percentage of regressions); (ii) articulation during oral reading negatively influenced the intraindividual consistency of reading performances with regard to a significant number of eye movement parameters; (iii) reading skills assessed by the LGVT reliably predicted spatial and temporal characteristics of forward eye movements, but not regressions when reading silently. When reading orally, reading skills were predictive of saccadic amplitudes, but not of the other parameters assessed (see section 4.1. on limitations of the study); (iv) for a restricted set of parameters, differences between silent and oral reading increased with reading skills; and (v) overall preschool cognitive abilities significantly predicted LGVT reading skills in adolescence, but not oculomotor control of either silent or oral reading. However, correlation coefficients indicated a small number of significant associations between performances on the individual subtests of the K-ABC and eye movements during oral reading, but less so during silent reading.

Compared to results reported for adult English readers (e.g., [14,28,29]), we found some quantitative differences in our sample of adolescent German readers: fixation durations observed in the present study were shorter for both silent and oral reading and the difference between reading modes was smaller; mean saccadic amplitudes (equaling an average length of 6.6 letters during silent and 6.0 letters during oral reading) were shorter; and the percentage of regressions was somewhat higher. As opposed to English readers in grades 1–5 [26] in our sample of adolescent German readers the percentage of regressions differed only marginally between silent and oral reading. Note that we observed remarkable interindividual differences for the percentage of regressions (10–40% during silent reading, 10–35% during oral reading), which were neither associated with reading skills nor with total text reading time. However, with respect to the association between individual reading skills and the percentage of leftward eye movements, previous research has produced inconsistent results for readers of more regular orthographies (e.g., [10,17,31,32,34–36]). As suggested by Rayner [14,28], eye movement parameters are not only influenced by orthographic regularity, but also by factors such as the complexity or difficulty of texts and characteristics of font. Thus, we would like to stress that such comparisons need to be interpreted with caution, in particular when differences in difficulty and complexity of a text may have an influence. Comprising multiple compounds (a characteristic of German) and biological terms, the text used in this study was rather difficult, which may have contributed to the differences outlined above.

Anderson and Swanson [23] also reported the difference between silent and oral reading to increase with reading skills: it was most significant in the group of good readers, followed by unselected readers, and smallest among poor readers. We focused on normal readers and decided against categorization into distinct groups. The use of metric data allowed us to apply regression analyses to define the relation of normal reading skills to the difference between silent and oral reading patterns. We found the difference between the two reading modes to increase with reading skills with respect to the following parameters: gaze duration, refixation time, number of fixations per word (marginal), total number of saccades, mean saccadic amplitude. Like Anderson and Swanson [23], we observed no significant association between reading skills and the difference in fixation duration. In contrast to their results, however, regressive eye movements did not vary according to reading skills.

With respect to correlations between eye movement parameters across reading modes, Anderson and Swanson [23] reported coefficients ranging from .50 and .78 across all groups of readers. Although in our study coefficients for six parameters were comparable, five coefficients failed to reach significance, indicating low levels of intraindividual consistency for at least a subset of basic eye movement parameters.

In line with studies reporting on the association of cognitive abilities and achievements in the speech-language domain (e.g., [6,42–44]), our results suggest preschool cognitive performances to predict later reading speed and reading comprehension assessed by means of the LGVT. Although we did not observe a statistically significant predictive power of K-ABC performance on eye movement patterns during either silent or oral reading, our study implies that early sequential and simultaneous processing performances might be–at least to some extent–related to eye movement patterns during oral reading. Interestingly, there were no significant correlations between sequential processing skills and eye movement parameters during silent reading, that is, the ability to serially and temporally organize a set of stimuli might be somehow related to the verbalization of written content.

### 4.1. Limitations

One limitation of the study is the rather small sample size. Participants were recruited for a longitudinal study right after birth and were followed up until the age of 13;6 (years;months). The drop-out rate was about 60%.

Another limitation is the assessment of reading skills. First, the LGVT being a test of silent reading skills, the closer relation between reading skills and eye movements assessed for silent as compared to oral reading (see section 3.4.) was as hypothesized. Perhaps we would have obtained a different pattern of results had we applied an assessment of oral reading skills. Second, the possibilities of interpreting the unique effects of reading speed and reading comprehension on oculomotor behavior are restricted. Reading speed and reading comprehension scores were calculated based on a single assessment and tended to be closely related in our sample (*r* = .76). Although common methods did not indicate multicollinearity, beta weights need to be interpreted with caution, as correlation coefficients indicated that in some equations one of the predictors was not additionally credited despite sharing a substantial amount of variance with the criterion.

A third limitation of our study refers to methodological aspects of the eye-tracking assessment: no clear instruction was given to the participants how they should deal with reading errors; the sequence of reading tasks was not randomized; and text comprehension not assessed. Thus, we cannot rule out a potential influence of sequence effects on our findings as oral reading was preceded by the silent reading task, the LGVT, as well as a series of neuromotor assessments (see section 2.4). We did not observe any signs of fatigue in our participants before or during the oral reading task, but a time-on-task effect or the increased predictability of the reading material (caused by the lack of counterbalancing) could have affected the differences between silent and oral reading.

### 4.2. Conclusion

The present study assessed basic characteristics of the oculomotor behavior of 21 adolescents during silent and oral text reading. We aimed to close the gap of missing comparisons of eye movements across reading modes in orthographically consistent languages.

In line with previous research (e.g., [23–27]; for reviews, see [14,28,29]), the results of the present study suggest that eye movement parameters differ substantially between silent and oral reading, and that eye movement parameters differ with respect to their consistency across reading modes. Furthermore, we found the association between individual reading skills and eye movement patterns to be higher during silent compared to oral reading, while preschool cognitive abilities were more closely associated with oral reading performances.

Although the overall pattern was the same as that of adult English-speaking cohorts, we found some quantitative differences. Future research might focus on potential developmental trends with regard to differences between silent and oral reading across different populations such as children, adults and the elderly, as well as normal and pathological readers.
